# A Functional Genetic Variant at the C-Reactive Protein Promoter (rs3091244) Is Not Associated With Cancer Risk in a Chinese Population

**DOI:** 10.3389/fimmu.2020.00926

**Published:** 2020-05-14

**Authors:** Ming-Yu Wang, Hai-Hong Zhou, Chun-Miao Zhang, Hai-Xiang Su, Shuo-Lei Li, Shang-Rong Ji, Enqi Liu, Yi Wu

**Affiliations:** ^1^Translational Medicine Research Center, MOE Key Laboratory of Cell Activities and Stress Adaptations, School of Life Sciences, Lanzhou University, Lanzhou, China; ^2^Children's Research Institute, Gansu Provincial Cancer Hospital, Lanzhou, China; ^3^MOE Key Laboratory of Environment and Genes Related to Diseases, School of Basic Medical Sciences, Xi'an Jiaotong University, Xi'an, China; ^4^The Affiliated Children's Hospital of Xi'an Jiaotong University, Xi'an, China

**Keywords:** cancer, inflammation, C-reactive protein, genetic variants, cancer risk

## Abstract

**Background:** The association of genetically elevated levels of circulating C-reactive protein (CRP) with cancer risk has been extensively investigated in European populations; however, there are conflicting conclusions. The tri-allelic rs3091244 is a functionally validated genetic variant, and its allelic frequencies differ significantly between European and Asian populations. Here, we examined the association of rs3091244 with cancer risk in a Chinese population.

**Methods:** rs3091244 was genotyped by Sanger sequencing in 4,971 cancer cases and 2,485 controls. The rs1205 and rs2794521 gene variants were also genotyped using TaqMan assays in subgroups.

**Results:** No association was detected between the genotyped CRP variants and cancer risk, with or without distinguishing cancer types, suggesting that circulating CRP is not causally involved in tumorigenesis in Chinese populations.

## Introduction

Genome instability and inflammation underlie the initiation and progression of cancer (1, 2). C-reactive protein (CRP) is a well-established circulating marker of inflammation (3) whose blood levels are positively associated with the risk and prognosis of several types of cancer (4, 5). Moreover, CRP has been shown to prevent the apoptosis of myeloma cells (6), to facilitate the invasiveness of breast cancer cells (7, 8), and to promote malignant properties of pancreatic neuroendocrine neoplasm cells (9). These findings raise the possibility that CRP, a putative soluble pattern recognition receptor acting in host defense and inflammation (10–16), might play a causal role in tumorigenesis.

A causal involvement suggests that genetically elevated levels of circulating CRP would affect cancer risk. However, large-scale genetic epidemiological studies (> 500 cases) fail to reach consistent conclusions, although European populations have mostly been examined (17–24). This might be explained by the fact that the examined genetic variants were not functionally linked to CRP expression. In addition, allelic frequencies of CRP variants usually differ among races. Therefore, examining whether functional CRP variants are associated with cancer risk in different populations might help to clarify the role of CRP in tumorigenesis.

Of the known CRP variants, only the promoter single-nucleotide polymorphism rs3091244, frequently observed in European and Asian populations, has been formally validated as a functional regulator of CRP expression (25–28). However, genotyping the tri-allelic rs3091244 is not trivial using the regular TaqMan assay. Thus, we genotyped this variant using Sanger sequencing and examined its association with the risk of any and specific types of cancer in a Chinese population. Our results revealed that rs3091244 and another promoter variant, rs2794521, were not associated with cancer risk, arguing for a noncausal role of circulating CRP in tumorigenesis.

## Materials and Methods

### Participants

Control and cancer cases are all Han Chinese. The types of cancer were diagnosed according to the criteria of WHO Classification of Tumors. Genomic DNA samples from cancer cases were obtained from the Tissue Bank of Gansu Cancer Hospital (diagnosed during 2015-2017). Genomic DNA samples from controls were obtained from individuals receiving health checks at Gansu Cancer Hospital (2016-2017). Informed consent for blood sampling was obtained by all participants. Their clinical characteristics are shown in Table 1 and Table S1. The study was approved by the Ethics Committee of the Gansu Provincial Cancer Hospital (A201307050027) and Xi'an Jiaotong University (2016-065) and was performed in accordance with relevant guidelines and regulations of the Ethics Committee.

**Table 1:** Characteristics of controls and cancer cases.

	**Control (2485)**	**All cancer (4971)**		**Gastric cancer (1557)**		**Breast cancer (1153)**	
		**Cases**	P^*^	**Cases**	P^*^	**Cases**	P^*^
Female sex, *n*	1125	2162	0.1513	361	<0.0001	1144	<0.0001
(%, total)	(45.27, 2485)	(43.49, 4971)		(23.19, 1557)		(99.22, 1153)	
Age, years, median	32	58	<0.0001	58	<0.0001	49	<0.0001
(IQR, total)	(27-39, 2485)	(49-65, 4971)		(50-65, 1557)		(43-56, 1153)	
BMI, kg/m^2^, median	22.98	22.26	<0.0001	21.25	<0.0001	23.82	0.0062
(IQR, total)	(20.69-25.47, 1552)	(20.07-24.76, 4635)		(19.33-23.73, 1439)		(21.77-26.07, 1114)	
CRP, mg/L, median	0.44	1.54	<0.0001	1.6	<0.0001	0.57	<0.0001
(IQR, total)	(0.15-0.95, 2046)	(0.40-9.08, 4778)		(0.38-8.94, 1489)		(0.20-1.52, 1127)	
Prior radio/chemotherapy, *n*		1124		220		364	
(%, total)		(40.23, 2794)		(29.69, 741)		(44.55, 817)	
Tumor Stage 0–2, *n*		1506		340		569	
(%, total)		(42.84, 3515)		(31.60, 1076)		(59.83, 951)	
	**Control (2485)**	**Lung cancer (1016)**		**Esophagus cancer (688)**		**Colorectal cancer (498)**	
		**Cases**	P^*^	**Cases**	P^*^	**Cases**	P^*^
Female sex, *n*		324	<0.0001	98	<0.0001	201	0.0481
(%, total)		(31.89, 1016)		(14.24, 688)		(40.36, 498)	
Age, years, median		60	<0.0001	63	<0.0001	59	<0.0001
(IQR, total)		(52-67, 1016)		(58-69, 688)		(50-66, 498)	
BMI, kg/m^2^, median		22.54	0.0071	21.3	<0.0001	22.4	0.0047
(IQR, total)		(20.51-24.74, 964)		(19.37-23.63, 641)		(20.06-24.91, 435)	
CRP, mg/L, median		5.95	<0.0001	2.46	<0.0001	2.08	<0.0001
(IQR, total)		(1.12-24.65, 978)		(0.57-12.64, 662)		(0.57-10.27, 474)	
Prior radio-chemotherapy, *n*		295		128		112	
(%, total)		(54.63, 540)		(36.06, 355)		(34.67, 323)	
Tumor Stage 0–2, *n*		132		295		167	
(%, total)		(22.88, 577)		(56.08, 526)		(45.26, 396)	

* *p values were determined by Fisher's exact test (for sex) or Wilcoxon's Rank Sums test (for age and BMI). IQR represents interquartile range. p value for CRP levels was determined with age correction by Scheirer-Ray-Hare test*.

### Genotyping

The tri-allelic single-nucleotide polymorphism rs3091244 was genotyped by Sanger sequencing with specific primers (forward: 5′ -AGGGGGGAGGGATAGCATTAGAA-3′; reverse: 5′ -CGTCCTGCTGCCAGTGATACAAG-3′) (BGI, Shenzhen, China). The bi-allelic single-nucleotide polymorphisms rs1205 and rs2794521 were genotyped using the TaqMan assay (Thermo Fisher Scientific, Rockford, IL; catalog number: c_7479334_10/c_318207_10; lot number: p151028-003).

### Luciferase Reporter Assay

The promoter fragment of CRP (−533~+103 bp) was cloned into the PGL4.10 (luc2) vector (Promega, Madison, WI; catalog number: E6651). Hep3B or HEK293T cells were transfected with 1.5 μg of PGL4.10 CRP reporter vector and 0.075 μg of phRL-TK (Promega; catalog number: E6241) using the X-treme GENE 9 DNA Transfection Reagent (Roche, Basel, Switzerland; catalog number: 06365787001; lot number: 23644700). After 48 h of transfection, luciferase activity was measured using the Dual-Luciferase Reporter Assay System (Promega; catalog number: E1960; lot number: 0000201344) on a Synergy HTX Multi-Mode Microplate Reader (BioTek, Winooski, VT). Firefly luciferase activities were normalized to that of co-transfected Renilla luciferase.

### Statistical Analysis

Hardy-Weinberg equilibrium was checked in healthy controls using the chi-squared test. Clinical characteristics between cases and controls were compared using Fisher's exact test, Wilcoxon signed rank test or Scheirer-Ray-Hare test. The activity of different alleles in the luciferase assay was tested using analysis of variance (ANOVA). The association of genotypes with circulating CRP levels was tested using a Kruskal-Wallis ANOVA. The crude odds ratio (OR) was estimated using a conditional logistic regression model to assess associations between CRP genotypes and cancer risk. OR trends among genotypes were calculated using the Cochran-Armitage trend test. *P* values below 0.05 were considered significant. Statistical analyses were conducted using SAS 9.3 (SAS Institute, Cary, NC, USA) or R package 3.6.0.

## Results

**Figure 1:**
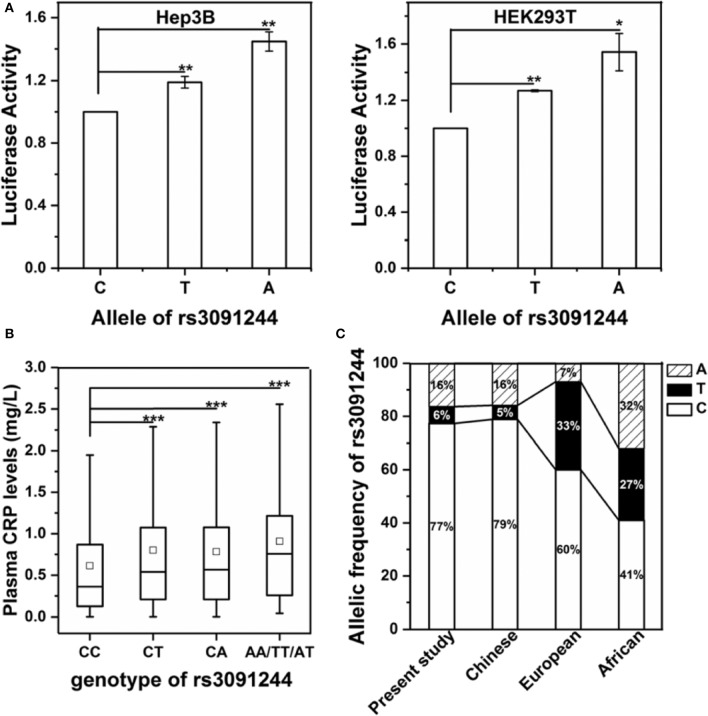
Validation of the functional impact of rs3091244 on CRP expression. **(A)** The effects of different rs3091244 alleles on CRP promoter activities in Hep3B (left) and HEK293 cells (right). The A- and T-alleles show higher luciferase reporter activities than the C-allele. **(B)** Serum levels of CRP in healthy controls with different genotypes of rs3091244 (CC: 1250, CT: 199, CA: 500, AA/TT/AT: 97). **(C)** Allelic frequencies of rs3091244 in the present study versus those in Chinese, European, and African populations obtained from the 1000 genomes project database. *p < 0.05; **p < 0.01; ***p < 0.001.

We first examined the direct effects of rs3091244 on the expression of human CRP using a luciferase reporter assay (Figure 1A). The promoter activity of human CRP increased in order with the rs3091244 C-, T- and A-alleles in both human hepatic Hep3B and renal HEK293 cells. This order also corresponded to levels of circulating CRP in healthy controls with different rs3091244 genotypes (Figure 1B). Overall, our results are in line with previous findings that rs3091244 functionally influences CRP expression (18, 25–27).

**Figure 2:**
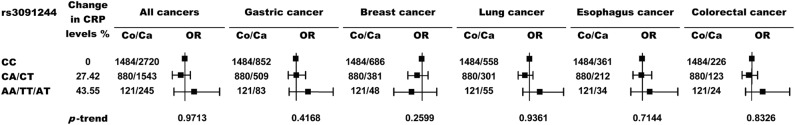
Risk of cancer by rs3091244 genotypes. *p* values (two-sided) are from a test for the trend of ORs across rs3091244 genotypes with increasing levels of circulating CRP. Black squares indicate ORs, and error bars indicate 95 % confidence intervals (CI). The numbers of control (Co) and cancer cases (Ca) are indicated.

Next, we genotyped the rs3091244 variant in 2485 healthy controls and 4971 cancer patients (Table 1 and Table S1). rs3091244 allelic frequencies in healthy controls were in Hardy-Weinberg equilibrium and were comparable to those in Asian populations, but differed from those in European populations (Figure 1C). Although the rs3091244 T- and A- alleles were associated with higher baseline levels of circulating CRP, they, either alone or in combination, showed no association with the risk of any type of cancer examined (Figure 2).

**Figure 3:**
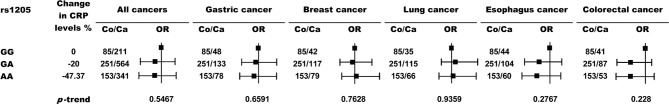
Risk of cancer by rs1205 genotypes. *P* values (two-sided) are from a test for the trend of ORs across genotypes with increasing levels of circulating CRP. Black squares indicate ORs, and error bars indicate 95 % confidence intervals (CI). The numbers of control (Co) and cancer cases (Ca) are indicated.

**Figure 4:**
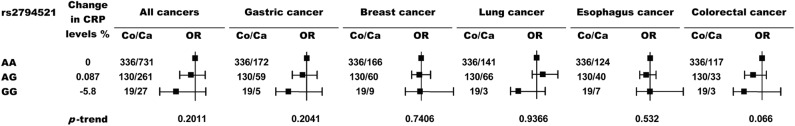
Risk of cancer by rs2794521 genotypes. *P* values (two-sided) are from a test for the trend of ORs across genotypes with increasing levels of circulating CRP. Black squares indicate ORs, and error bars indicate 95 % confidence intervals (CI). The numbers of control (Co) and cancer cases (Ca) are indicated.

**Figure 5:**
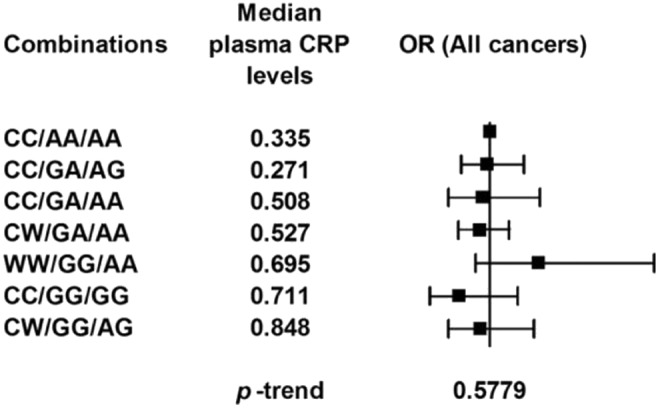
Risk of cancer by rs3091244, rs1205, and rs2794521 genotypes. The genotype combinations of the three single-nucleotide polymorphisms are given in the order of rs3091244/rs1205/rs2794521. W represents the A or T allele of rs3091244. *P* values (two-sided) are from a test for the trend of ORs across genotypes with increasing levels of circulating CRP. Black squares indicate ORs, and error bars indicate 95 % confidence intervals (CI).

We also genotyped the most frequently examined CRP variant, rs1205, and another CRP promoter variant, rs2794521, in a subgroup of 489 healthy controls and 1116 cancer patients using TaqMan assays. However, these two variants (Figures 3, 4) or their combination with rs3091244 (Figure 5) again showed no association with cancer risk. Thus, we propose that circulating CRP is unlikely to be casually involved in tumorigenesis in Chinese populations.

## Discussion

There have been several large-scale studies investigating the associations between genetic CRP variants and cancer risk in European (17–21, 24), American (22) and Chinese populations (23). One study claimed that CRP variants are not associated with the overall cancer risk (18), while others reported the opposite (19) and also identified risk associations with certain cancer types (17, 19, 20, 22). Confusingly, the identified variants can be associated with increased cancer risk but with decreased levels of circulating CRP (17, 22). Such a negative association is also observed for a different cancer type, i.e., breast cancer, in the present study albeit statistically insignificant. Nevertheless, these were difficult to reconcile with the established positive association between cancer risk and circulating CRP levels. Moreover, the same CRP variant rs1205 has been found to be associated with an increased risk of colon cancer in one study (22) but with decreased risk in another (20).

Regarding these inconsistencies, it should be noted that a clear functional association with CRP expression has not been demonstrated for most examined variants (17–23). The only functional variant, rs3091244 (25–27) was only examined in one study in the general population of Denmark (18). This might partly be due to the tri-allelic nature of rs3091244, which is difficult to be genotyped using the regular TaqMan assay (18). The present study used the more accurate Sanger sequencing to genotype rs3091244 in a Chinese population, and reached the same conclusion as the Denmark study (18). The lack of a significant association between rs3091244 and cancer risk suggests that circulating CRP does not play a causal role in tumorigenesis.

The noncausal involvement of circulating CRP in cancer was not entirely unexpected. Our recent work has revealed that tissue-localized CRP may be predominantly produced *in situ* rather than transported from the circulation (29). This would imply that instead of liver-produced, circulating CRP, it is locally-produced CRP that potentially plays an etiological role in tumorigenesis. We have further shown that malignant cell-derived CRP can conversely be transported to the circulation (29), likely contributing to the risk-associated subtle elevations of circulating CRP. However, such contributions might be obscured in genetic association studies due to confounding factors including tissue-specific effects of genetic variants on CRP expression, and their profound modulation on circulating CRP levels. Whether extrahepatic tissue-derived CRP is causally involved in tumorigenesis remains to be investigated.

## Data Availability Statement

The datasets generated for this study can be found in the article/Supplementary Material.

## Ethics Statement

The studies involving human participants were reviewed and approved by the Ethic Committee of the Gansu Provincial Cancer Hospital (A201307050027) and Xi'an Jiaotong University (2016-065). Written informed consent to participate in this study was provided by the participants' legal guardian/next of kin.

## Author Contributions

YW, EL, and S-RJ designed the research. M-YW, H-HZ, C-MZ, H-XS, and S-LL performed the research. YW, EL, M-YW, and H-HZ analyzed the data and wrote the paper. All authors reviewed the results and approved the final version of the manuscript.

## Conflict of Interest

The authors declare that the research was conducted in the absence of any commercial or financial relationships that could be construed as a potential conflict of interest.
